# Inhibition of Poly(ADP-ribose) Polymerase-1 Enhances Gene Expression of Selected Sirtuins and APP Cleaving Enzymes in Amyloid Beta Cytotoxicity

**DOI:** 10.1007/s12035-017-0646-8

**Published:** 2017-07-12

**Authors:** Przemysław L. Wencel, Walter J. Lukiw, Joanna B. Strosznajder, Robert Piotr Strosznajder

**Affiliations:** 1 Laboratory of Preclinical Research and Environmental Agents, Department of Neurosurgery, Mossakowski Medical Research Centre, Polish Academy of Sciences, Pawińskiego 5, 02-106 Warsaw, Poland; 2 LSU Neuroscience Center, Louisiana State University Health Sciences Center, 2020 Gravier Street, Suite 904, New Orleans, LA 70112 USA; 3 Department of Cellular Signalling, Mossakowski Medical Research Centre, Polish Academy of Sciences, Pawińskiego 5, 02-106 Warsaw, Poland

**Keywords:** Poly(ADP-ribose) polymerases, Sirtuins, Amyloid, Alzheimer’s disease, APP, PARPs, SIRTs, Secretases, Neuroprotection

## Abstract

Poly(ADP-ribose) polymerases (PARPs) and sirtuins (SIRTs) are involved in the regulation of cell metabolism, transcription, and DNA repair. Alterations of these enzymes may play a crucial role in Alzheimer’s disease (AD). Our previous results indicated that amyloid beta (Aβ) peptides and inflammation led to activation of PARP1 and cell death. This study focused on a role of PARP1 in the regulation of gene expression for SIRTs and beta-amyloid precursor protein (βAPP) cleaving enzymes under Aβ42 oligomers (AβO) toxicity in *pheochromocytoma* cells (PC12) in culture. Moreover, the effect of endogenously liberated Aβ peptides in PC12 cells stably transfected with human gene for APP wild-type (APPwt) was analyzed. Our results demonstrated that AβO enhanced transcription of presenilins (*Psen1* and *Psen2*), the crucial subunits of γ-secretase. Aβ peptides in APPwt cells activated expression of β-secretase (*Bace1*), *Psen1*, *Psen2*, and *Parp1*. The inhibitor of PARP1, PJ-34 in the presence of AβO upregulated transcription of α-secretase (*Adam10*), *Psen1*, and *Psen2*, but also *Bace1*. Concomitantly, PJ-34 enhanced mRNA level of nuclear *Sirt1*, *Sirt6*, mitochondrial *Sirt4*, and *Parp3* in PC12 cells subjected to AβOs toxicity. Our data indicated that Aβ peptides through modulation of APP secretases may lead to a vicious metabolic circle, which could be responsible for maintaining Aβ at high level. PARP1 inhibition, besides activation of nuclear SIRTs and mitochondrial *Sirt4* expression, enhanced transcription of enzyme(s) involved in βAPP metabolism, and this effect should be considered in its application against Aβ peptide toxicity.

## Introduction

Among all neurodegenerative disorders, Alzheimer’s disease (AD) is the most severe dementia that progressively and irreversibly impairs synaptic function and cognition [1–3]. Until now, the pathogenesis and pathomechanisms of AD is unknown, and no therapy to date has proven to be successful. For the last two decades, the amyloid hypothesis dominated in research field on AD [4–6].

Although the hypothesis of “pathogenic spread” of amyloid beta (Aβ) peptides in neurodegenerative disease has been widely suggested, recent studies have provided evidences for human transmission of amyloid β pathology and cerebral amyloid angiopathy [7, 8].

Aβ peptides are released from beta-amyloid precursor protein (βAPP) which is a membrane protein involved in regulation of synapse formation, neuronal growth, and repair [9, 10]. βAPP is metabolized by two separate pathways: non-amyloidogenic and amyloidogenic. In the non-amyloidogenic pathway, α- and γ-secretases are responsible for βAPP degradation to peptide p3 and αAPP. Moreover, several zinc metalloproteinases, including ADAM10, can mediate βAPP cleavage at α-secretase sites [11].

On the contrary, in the amyloidogenic pathway, βAPP is degraded at β-site by beta secretase (BACE1) and then by γ-secretase to yield Aβ peptides [12, 13]. γ-secretase is a tetrameric protein complex with presenilins (PSEN1 and PSEN2) as crucial components involved in processing at both α- and β- C-terminal fragment sites [14, 15]. The most common causes of familial AD are mutations of genes encoding presenilins 1 and 2 and β-secretase which lead to increased production of highly amyloidogenic Aβ42 isoform [16, 17].

Currently, among all Aβ forms oligomers (AβO) are suggested to be the most toxic and correlated with dementia [18]. Several studies have indicated that the most toxic are Aβ peptide dimers and probably trimers isolated from Alzheimer brains, and this molecular species may impair memory [19]. Independently of which types of Aβ oligomers are responsible for disrupting cognition, the question arises—what kind of molecular processes altered by Aβ peptides are responsible for dementia and cell loss and how to protect cells against Aβ toxicity?

It is generally accepted that oxidative stressors play a crucial role in pathomechanism of neurodegenerative diseases. Oxidative stress evoked by Aβ peptides may lead to disturbance of many key processes involved in regulation of cell viability and death [20, 21]. Mitochondria were recently indicated to be the target for APP that accumulates in the mitochondrial import channels and for Aβ that interacts with several proteins inside mitochondria [22–30]. Among several pro- and anti-oxidative enzymes, the most important stress response proteins are both NAD^+^ dependent enzymes families such as the following: sirtuins (SIRTs) and poly(ADP-ribose) polymerases (PARPs). These enzymes depending on stress conditions may exert neuroprotective effect or may lead to cells degeneration and death. The relationship between PARPs and SIRTs may play important role in regulation of cells’ fate [31–33].

SIRTs are a family of seven enzymes that are deacetylases [type III histone deacetylases (HDAC’s)], and some of them are ADP-ribosyltransferases. Three of them (SIRT3, SIRT4, SIRT5) primarily exist in mitochondria. The mitochondrial SIRTs can modulate the activity of antioxidative enzymes, and they can protect cell against oxidative stress by direct stimulation of expression and activity of Mn superoxide dismutase (SOD_2_) or indirectly by activation of glutathione peroxidase (GPx) and by enhancing mitochondrial electron transport activity [34, 35]. Mitochondrial SIRT3 and nuclear SIRT1 are the most well-studied SIRTs. They are also widely expressed in brain and other tissues that have high metabolic activity [36–39]. Sirtuins can interact with PARPs, and all these enzymes are involved in regulation of transcription, DNA repair, cell metabolism, survival, and death. PARPs are capable transfer and polymerization of ADP-ribose units using NAD^+^ molecules. PARP activity results in the attachment of linear or branched polymers of ADP-ribose to target proteins (heteromodification) or to PARP itself (automodification). PARP1 is responsible for over 90% of poly(ADP-ribosylation) in the brain [40–42]. Among the family, PARP1, PARP2, and PARP3 have been found to be induced by DNA strand breaks, and their role in DNA repair process has been reviewed [43, 44]. In AD and other neurodegenerative disorders, accumulation of Aβ in the central nervous system may induce neuronal cell death accompanied by overexpression of PARP1 [30, 45–50]. Overactivation of PARP1 may lead to the NAD^+^ depletion, followed by inhibition of SIRT1 activity. Another recent study revealed protective effects of PARP1 inhibitors on oxidative stress and mitochondrial integrity in an ex vivo model of AD induced by Aβ42 [51]. Moreover, in response to sustained stress, PARP1 can be overactivated and in consequence may alter the function of many transcription factors and other proteins including the following: p53, NF-κB, AIF, HIF-1, FOXO1, Bax, and Bcl-2 [48, 49, 52–54]. The last data of Martire et al. [55] demonstrated that PARP1 inhibition rescued metabolic dysfunction, bioenergetics impairment, and restored inhibition of pyruvate kinase 2 expression in animal and cellular models of AD. Despite intensive studies during the last decade, the role of PARP1 in the regulation of nuclear and mitochondrial SIRTs and enzymes responsible for APP metabolism is not fully understood and elucidated.

In this study, we investigated the effect of PARP1 inhibition on gene expression of enzymes responsible for stress response such as NAD^+^ dependent nuclear *Sirt1*, *Sirt6*, cytosolic *Sirt2*, and mitochondrial SIRTs (*Sirt3*, *Sirt4*, *Sirt5*) in Aβ toxicity. Moreover, for a better understanding of the role of poly(ADP-ribosylation) in βAPP metabolism, the effect of PARP inhibitor on gene expression of βAPP degrading enzymes was analyzed.

## Materials and Methods

### Preparation of Aβ1–42 Oligomers

Aβ 1–42 (rPeptide no. A-1163-2) oligomers were prepared according to Stine et al. [56], as described previously by Cieslik et al. [20]. Amyloid β was dissolved (5 mM Aβ 1–42) in anhydrous DMSO and then diluted to 100 μM concentration with ice-cold cell culture medium (phenol red-free Ham’s F-12; BioSource), immediately vortexing for 30 s, and then incubated at 4 °C for 24 h. The same procedure was used for preparation of Aβ scrambled 1-42 (Aβscr) (rPeptide no. A-1004-2). Conformation state of Aβ was confirmed using Thioflavin T (ThT) which is benzothiazole dye binding to amyloid fibrils. Electrophoretic analysis of used Aβ42 followed by silver staining was performed.

### Cell Culture and Cell Treatment Protocol

Rat *pheochromocytoma* (PC12) cells were cultured in Dulbecco’s Modified Eagle’s Medium (DMEM) supplemented with 10% heat-inactivated fetal bovine serum (FBS), 5% heat-inactivated horse serum (HS), 2 mM L-glutamine, 50 U/ml penicillin, and 50 μg/ml streptomycin in a 5% CO_2_ atmosphere at 37 °C.

Moreover, PC12 cells transfected with human gene for APP wild-type (APPwt) was used. As a control, we used cells transfected with an empty vector. These cells were a kind gift from Prof Dr. W. E. Mueller and Prof A. Eckert from the University of Frankfurt and Basel. Transfected PC12 cells APPwt and control were cultured in the same medium as mentioned before with addition of selective antibiotic 400 μg/ml G-418 sulfate. Cell treatment was performed in serum-free Neurobasal medium supplemented with B27 (Gibco), 1% penicillin/streptomycin, and 2 mM L-glutamine in order to stop proliferation of cells. Then, the PC12 cells were treated with 1 μM Aβ 1–42 oligomers (AβO) and (20 μM) PJ-34 for 24 h.

The procedure of cell transfection was described previously [57], and these APP transfected cells were used by us in several studies [26, 58–60]. Moreover, APP transfected cells were investigated under EM [61].

### Determination of Cell Survival Using the MTT Test

Cell viability was evaluated by reduction of 2-(4,5-dimethylthiazol-2-yl)-2,5 diphenyltetrazolium bromide (MTT) to formazan. After 24 h of treatment with the tested compounds, MTT (2.5 mg/ml) was added to the wells. The cells were incubated at 37 °C for 2 h. Then, the medium was removed, the formazan crystals were dissolved in 150 μL DMSO, and absorbance was measured at 595 nm [20].

### Quantitative Real-Time PCR Assays

PC12 cells were washed twice with ice-cold PBS and suspended in 1 ml of TRI reagent (Sigma-Aldrich). RNA was isolated from the cell pellet according to the manufacturer’s protocol. Digestion of DNA contamination was performed by using DNase I according to the manufacturer’s protocol (Sigma-Aldrich). Reverse transcription was performed using a High Capacity cDNA Reverse Transcription Kit as described in the manufacturer’s protocol (Applied Biosystems, Foster City, CA, USA). The level of mRNA for selected genes was analyzed using TaqMan Gene Expression Assays (Applied Biosystems, Foster City, CA, USA) according to the manufacturer’s instructions. β-actin (*Actb*) was selected and used in all of the studies as a reference gene. Quantitative PCR was performed using an Applied Biosystems 7500 Real-Time PCR System. The following genes were analyzed: *Sirt1* Rn01428096_m1, *Sirt2* Rn01457502_m1, *Sirt3* Rn01501410_m1, *Sirt4* Rn01481485_m1, *Sirt5* Rn01450559_m1, *Sirt6* Rn01408249_m1, *Parp1* Rn00565018_m1, *Parp2* Rn01414610_m1, *Parp3* Rn01447502_m1, *Adam10* Rn01530753_m1, *Bace1* Rn00569988_m1, *Psen1* Rn00569763_m1, and *Psen2* Rn00579412_m1. As a reference, we used *Actb* gene Rn00667869_m1. The relative level of mRNA was calculated using the ∆∆Ct method.

### Statistical Analysis

All experiments were repeated at least three times and were performed either in triplicate or duplicate. The presented data are the means ± SEM. For statistical comparison, Student’s *t* test or one-way ANOVA followed by Newman-Keuls *post hoc* test were used. *P* values < 0.05 were considered statistically significant (*−p < 0.05; **−p < 0.01; ***−p < 0.001). The statistical analyses were performed by using Graph Pad Prism version 6.0 (Graph Pad Software, San Diego, CA, USA).

## Results

In the present study, we have investigated the role of PARP1 in regulation/alterations of gene expression for stress responses proteins such as NAD^+^ dependent SIRTs and DNA bound PARPs in PC12 cells under Aβ toxicity. Moreover, the effect of PARP1 on gene expression of APP metabolizing enzymes which regulate Aβ concentration was analyzed. The effect of exogenous Aβ42 on the transcription of these enzymes was compared with the action of endogenously liberated Aβ peptides in PC12 cells with overexpressed human APP.

### Description of Aβ42 Oligomers (AβO) and Their Role in Cell Viability

**Fig. 1:**
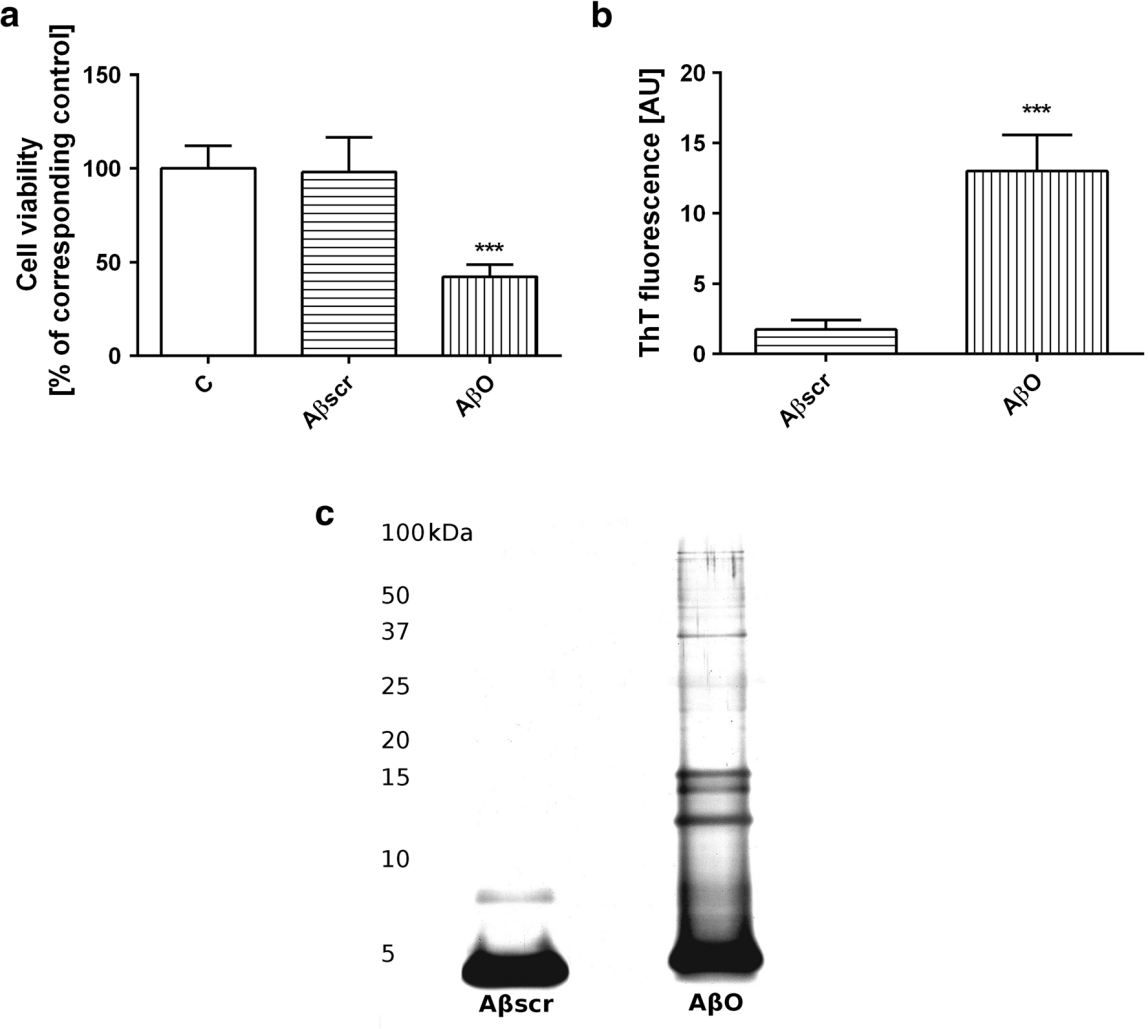
The effect of Aβ oligomers on PC12 cells viability. The effect of oligomeric Aβ42 or scrambled Aβ at 1 μM concentration on PC12 cell viability after 24-h treatment (**a**). Thioflavin (ThT) fluorescence analysis of the Aβ42 scrambled and Aβ42 in oligomeric form (**b**). Proteins were subjected to denaturing SDS–PAGE followed by silver staining. Electrophoretic analysis of the Aβ42 scrambled and Aβ42 in oligomeric form (AβO) (**c**). For statistical comparison, Student’s *t* test or one-way analysis of variance (ANOVA) with Neuman-Keuls *post hoc* test was used. *P* values <0.05 were considered statistically significant (***−*p* < 0.001)

First of all, we have investigated whether Aβ42 in oligomeric form (1 μM) affects PC12 cells survival. Our data demonstrated that AβO decreased cell viability by about 60% compared to the control: non-treated cells and Aβ scrambled (Aβscr) peptide (Fig. 1a). We observed that Aβ42 oligomers enhanced ThT fluorescence, but such effect was not seen with Aβscr (Fig. 1b). Moreover, Aβ oligomers were separated using denaturing SDS-PAGE followed by silver staining. The results have shown the presence of Aβ oligomers, trimers, and tetramers and only small amount of polymers (Fig. 1c).

### AβO and PARP-1 Inhibitor Alter Transcription of Sirtuins and DNA-Bound PARPs

**Fig. 2:**
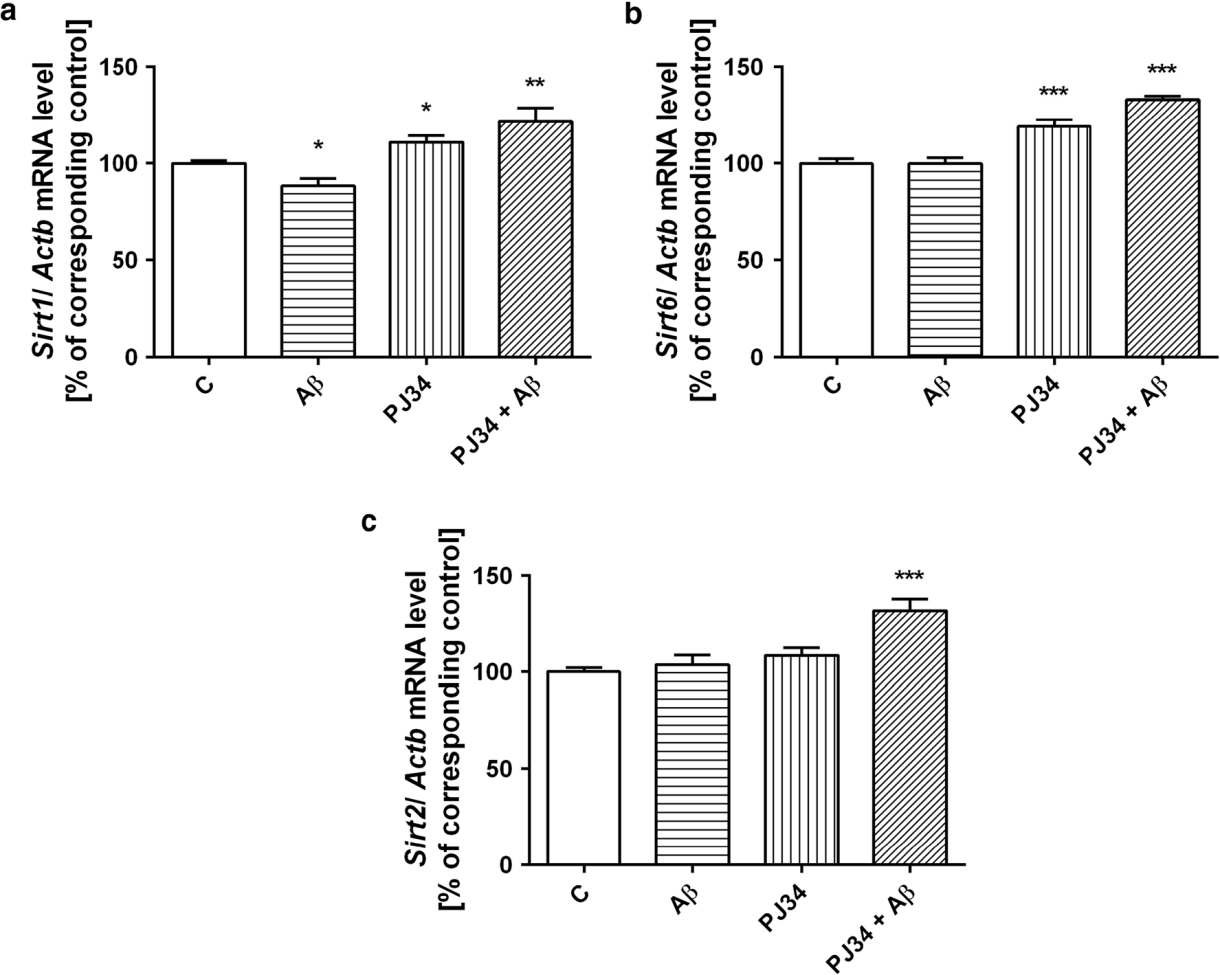
The effect of oligomeric Aβ42 and PJ-34 treatment on gene expression of nuclear and cytosolic sirtuins. PC12 cells were incubated in the presence of oligomeric Aβ42 (AβO, 1 μM) and PJ-34 (20 μM) for 24 h (**a**–**c**). The levels of mRNA of nuclear *Sirt1* (**a**), *Sirt6* (**b**), and cytosolic *Sirt2* (**c**) were analyzed via quantitative RT-PCR. The results of RT-PCR were normalized to *Actb* gene expression. Data represent the mean value ± SEM for three independent experiments with 3–4 replications. ***p < 0.001; **p < 0.01; *p < 0.05—the difference, that was statistically significant compared to the control cells, using one-way analysis of variance (ANOVA) with Neuman-Keuls *post hoc* test

**Fig. 3:**
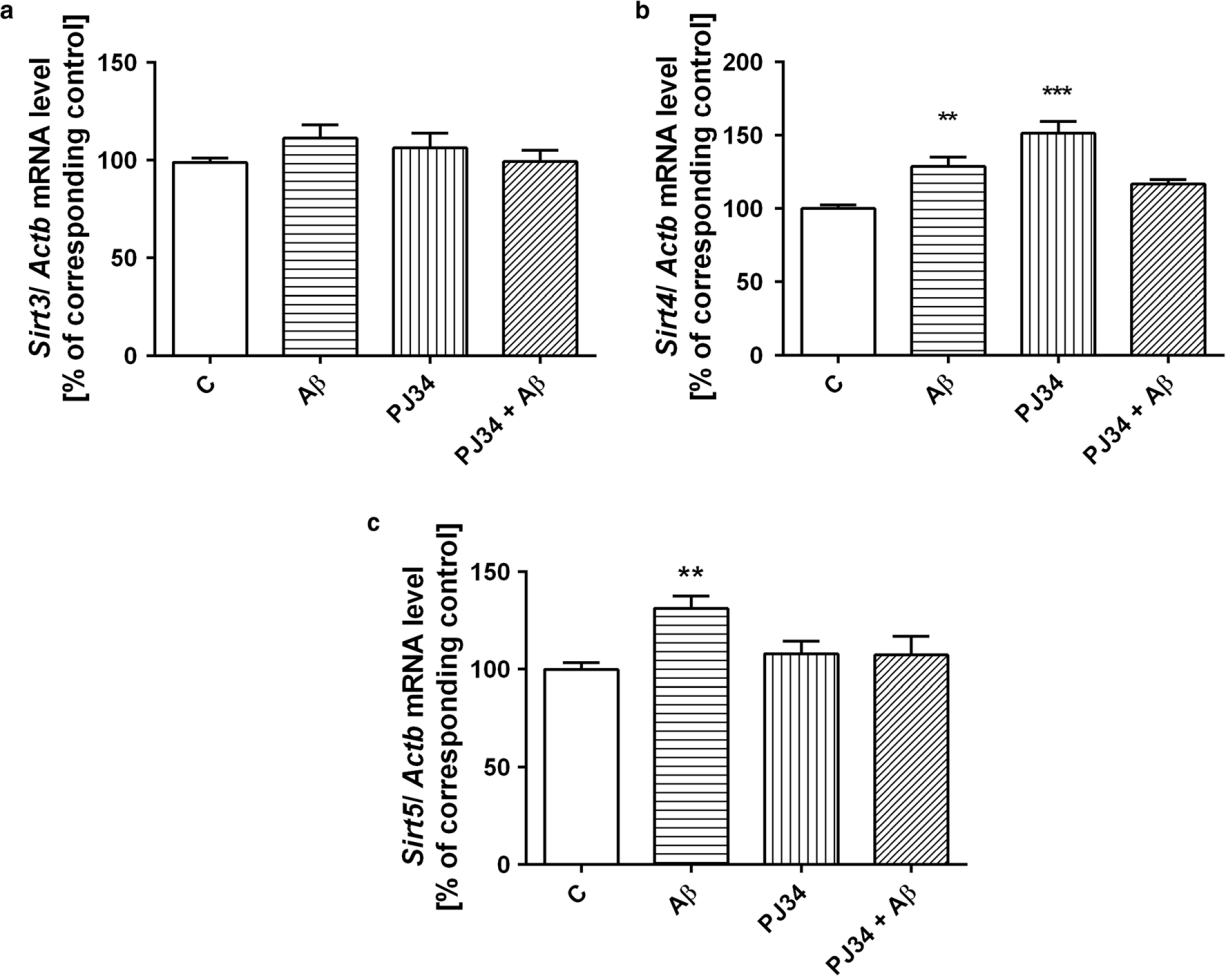
The influence of PARP1 inhibitor PJ-34 and oligomeric Aβ42 treatment on gene expression of mitochondrial sirtuins. PC12 cells were incubated in the presence of oligomeric Aβ42 (AβO, 1 μM) and PJ-34 (20 μM) for 24 h (**a**–**c**). The levels of mRNA of mitochondrial sirtuins—*Sirt3* (**a**), *Sirt4* (**b**), and *Sirt5* (**c**)—were analyzed via quantitative RT-PCR. The results of RT-PCR were normalized to *Actb* gene expression. Data represent the mean value ± SEM for three independent experiments with three replications. ***p < 0.001; **p < 0.01—the difference, that was statistically significant compared to the control cells, using one-way analysis of variance (ANOVA) with Neuman-Keuls *post hoc* test

In the following experiments, the pharmacological inhibition of PARP1 (using specific inhibitor PJ-34) significantly activated transcription of *Sirt1* and *Sirt6* (Fig. 2a, b). AβO significantly decreased *Sirt1* expression (Fig. 2a). AβO and PJ-34 acting separately had negligible stimulatory effect on cytosolic *Sirt2* expression, but PJ-34 acting in the presence of AβO exerted significant activation on *Sirt2* (Fig. 2c). AβOs influenced transcription of mitochondrial SIRTs with one exception of *Sirt3* where negligible stimulatory effect was observed (Fig. 3a). AβOs significantly enhanced gene expression of mitochondrial *Sirt4* and *Sirt5* (Fig. 3b, c). Moreover, PJ-34 upregulated gene expression of *Sirt4* (Fig. 3b). The further study demonstrated that AβOs had no significant effect on gene expression of *Parp1* and other DNA bound PARPs (Table 1). Inhibition of PARP1 activity led to activation of gene expression of *Parp3* in the absence and presence of AβO (Table 1).

**Table 1:** The effect of Aβ42 oligomers and PARP1 inhibitor on the level of mRNA of DNA bound PARPs

Gene	mRNA level of DNA bound PARPs/*Actb* [% of control]		
	Aβ	PJ-34	Aβ + PJ-34
*Parp1*	120.6 ± 9.10	106.40 ± 2.75	115.10 ± 0.95
*Parp2*	109.40 ± 5.31	100.40 ± 3.96	109.30 ± 6.23
*Parp3*	98.94 ± 5.60	118.5 ± 0.70*	130.00 ± 5.82*

PC12 cells were incubated in the presence of oligomeric Aβ42 (AβO, 1 μM) and PJ-34 (20 μM) for 24 h. The levels of mRNA of DNA bound PARPs—*Parp1*, *Parp2*, and *Parp3*—were analyzed via quantitative RT-PCR. The results of RT-PCR were normalized to *Actb* gene expression. Data represent the mean value ± SEM for three independent experiments with three replications. *p < 0.05—the difference, that was statistically significant compared to the control cells, using one-way analysis of variance (ANOVA) with Neuman-Keuls *post hoc* test

### AβO and PARP-1 Inhibitor Affect Genes Expression Involved in APP Metabolism

**Fig. 4:**
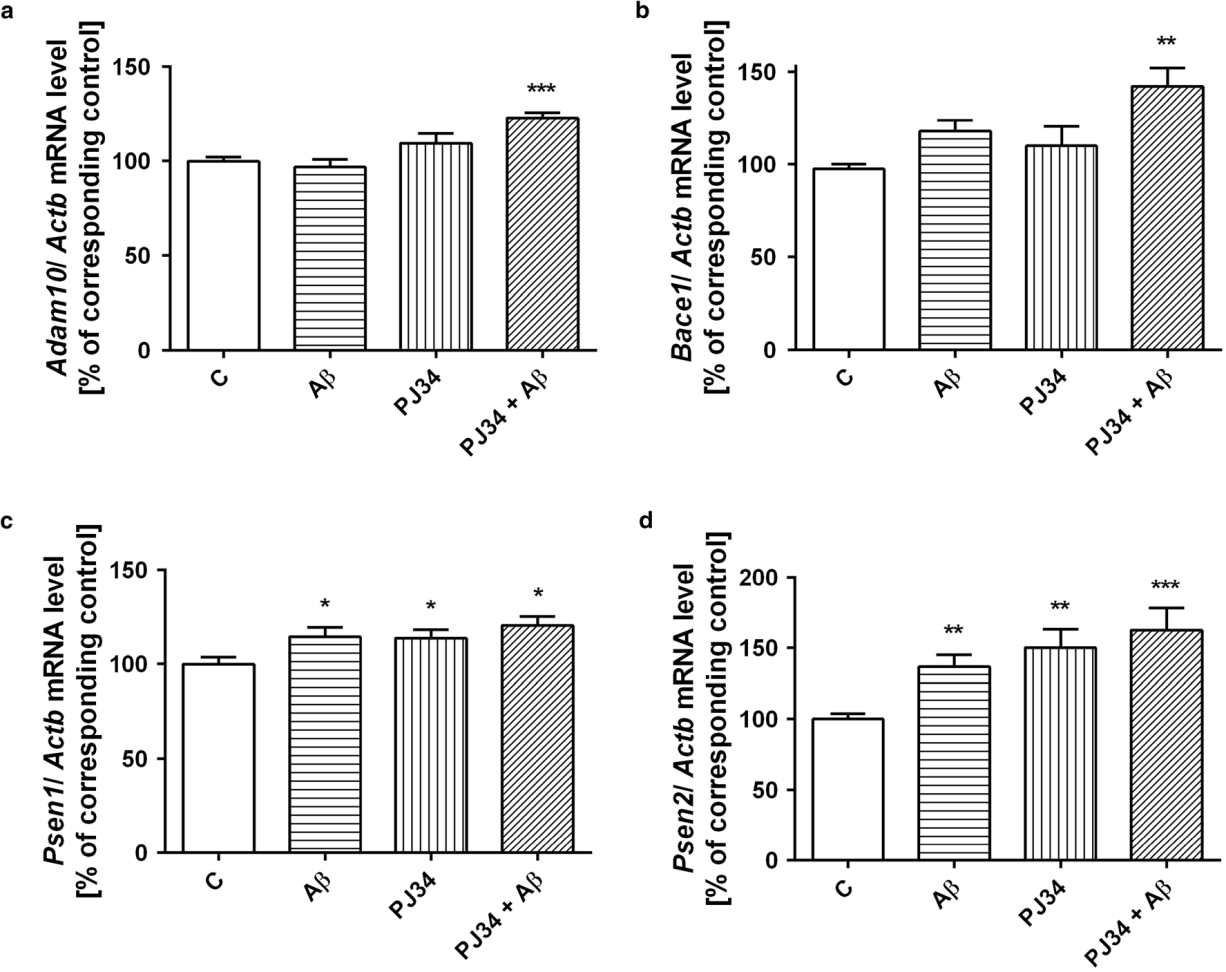
Expression levels of *Adam10*, *Bace1*, *Psen1*, and *Psen2* genes in PC12 cells subjected to AβO42 (1 μM) and PJ-34 (20 μM) treatments (**a**–**d**). The levels of mRNA of secretases—*Adam10* (**a**), *Bace1* (**b**), *Psen1* (**c**), and *Psen2* (**d**)—were analyzed via quantitative RT-PCR. The results of RT-PCR were normalized to *Actb* gene expression. Data represent the mean value ± SEM for three independent experiments with three replications. ***p < 0.001; **p < 0.01, *p < 0.05—the difference, that was statistically significant compared to the control cells, using one-way analysis of variance (ANOVA) with Neuman-Keuls *post hoc* test

Then, question arises how AβO modulates transcription of enzymes responsible for APP processing in amyloidogenic and non-amyloidogenic pathways. In this experimental setting, AβOs exerted no effect on α-secretase (*Adam10*) expression and also on gene expression of β-secretase (*Bace1*) (Fig. 4a, b). PJ-34 significantly activated expression of *Adam10* and *Bace1* in the presence of AβO (Fig. 4a, b). Analysis of AβOs action on APP processing indicated the significant stimulatory effect of AβOs on gene expression of *Psen1* and *Psen2* (Fig. 4c, d). Moreover, PJ-34 had also stimulatory effect on *Psen1/2* mRNA levels in the absence and presence of Aβ (Fig. 4c, d).

### The Role of Endogenous Aβ Peptides on Transcription of APP Secretases and NAD^+^ Dependent Enzymes

**Fig. 5:**
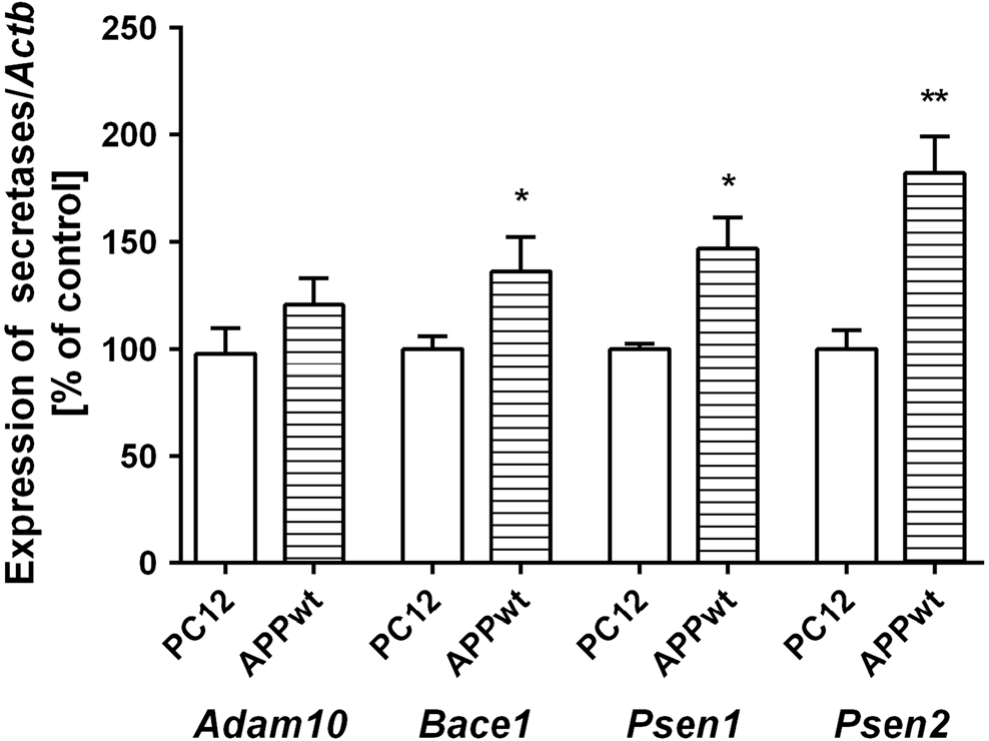
Expression levels of APP secretases in PC12 cells transfected with human gene for APP (APPwt) or with an empty vector. The levels of mRNA of secretases: *Adam10*, *Bace1*, *Psen1*, and *Psen2* were analyzed via quantitative RT-PCR. The results of RT-PCR were normalized to *Actb* gene expression. Data represent the mean value ± SEM for three independent experiments. **p < 0.01; *p < 0.05—the difference, that was statistically significant compared to the control cells, using a Student’s *t* test

**Fig. 6:**
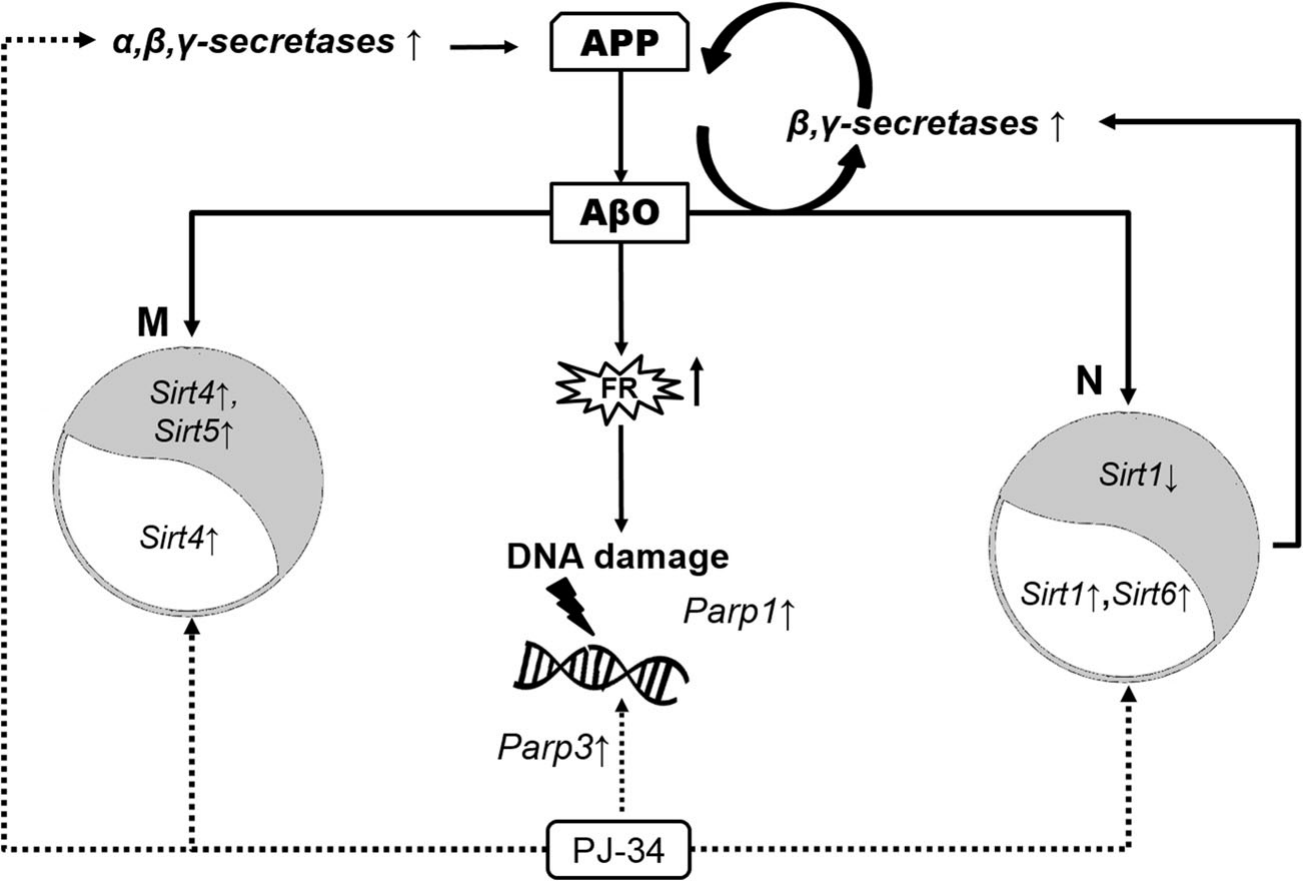
Interactions between Aβ oligomers and PARP1 inhibitor (PJ-34) on genes expression of sirtuins, DNA bound PARPs, and APP metabolizing enzymes. Effect of Aβ in mitochondria (M) and nucleus (N) is demonstrated on the *grey field*

The effect of exogenous Aβ42 oligomers was then compared with the action of endogenously liberated Aβ peptides in APPwt cells. In this APP transfected cells, significant activation of gene expression of *Bace1* and also of *Psen1* and *Psen2* was observed (Fig. 5). Although *Parp1* expression was enhanced, transcription of other DNA bound PARPs and mitochondria SIRTs was not changed (Table 2). The relationship between described molecular events evoked by Aβ peptides, and the effect of PARP1 pharmacological inhibition is demonstrated on Fig. 6.

**Table 2:** Gene expression and DNA bound PARPs and mitochondrial sirtuins in APPwt transfected cells

Gene	PARPs and SIRTs/*Actb* mRNA level [% of control]
*Parp1*	158.6 ± 2.42*
*Parp2*	106.4 ± 1.27
*Parp3*	91.4 ± 1.93
*Sirt3*	120.6 ± 2.93
*Sirt4*	74.43 ± 10.37
*Sirt5*	58.78 ± 37.22

The levels of mRNA of DNA bound PARPs—*Parp1*, *Parp2*, *Parp3*—and mitochondria SIRTs—*Sirt3*, *Sirt4*, *Sirt5*—were analyzed via quantitative RT-PCR. The results of RT-PCR were normalized to *Actb* gene expression. Data represent the mean value ± SEM for three independent experiments. *p < 0.05—the difference, that was statistically significant compared to the control cells, using a Student’s *t*-test

## Discussion

Our data demonstrate for the first time that PARP1 pharmacological inhibition by PJ-34 significantly enhances the expression of nuclear *Sirt1* and *Sirt6* in control cells and in Aβ42 toxicity. It was previously shown that in mammalian cells subjected to oxidative stress, SIRT1 and SIRT6 are responsible for DNA double-strand breaks repair [62–64]. Moreover, S*irt*6 knockout mice exhibit premature aging phenotype and disturbed base excision repair (BER). In our experimental conditions, activation of *Sirt1* and *Sirt6* expression suggests the significant involvement of these both nuclear SIRTs in oxidative stress evoked by Aβ toxicity and by PARP1 inhibition. The inhibition of PARP1 activates also gene expression of cytosolic *Sirt2* and mitochondrial *Sirt4*, however, remains without effect on *Sirt3* and *Sirt5*. Transcription of mitochondrial *Sirt4* and *Sirt5* is significantly enhanced by AβO. These both SIRTs could be responsible for the mitochondrial proteins modifications by several processes as mono ADP-ribosylation, succinylation, and sumolynation which may subsequently affect mitochondrial protein function. PARP inhibitor decreases *Sirt4* and *Sirt5* transcription elevated by AβO toxicity.

Moreover, our results indicated that PARP1 inhibition and AβOs lead to activation of genes coding enzymes responsible for APP metabolism. PARP1 pharmacological inhibition enhanced mRNA level of *Psen1* and *Psen2* crucial subunits of γ-secretase involved in degradation of APP. PARP1 inhibition in the presence of AβOs enhanced gene expression of α-secretase and in the same experimental condition exerted significant stimulatory effect on transcription of β-secretase. Endogenously liberated Aβ peptides in βAPP transfected cells were found to exert stimulatory effects on transcription of PSENs and also β-secretase.

These results suggest that the vicious toxic cycle between Aβ and βAPP metabolism may be responsible for high levels of neurotoxic C99 fragment and Aβ peptides in cells. Our previous results showed that oxidative/genotoxic stress evoked by Aβ, inflammation processes, and brain ischemia led to activation of PARP1 and accumulation of PAR [52, 65–68]. In this study, we have observed that Aβ enhanced significantly gene expression of *Parp1* in APPwt cells. Inhibitors of PARP are therefore suggested to be promising in protection of cells against neurodegeneration. They have stimulatory effect on gene expression of *Sirt1*, *Sirt6*, and non-amyloidogenic pathway which may exert cytoprotective effect. On the other hand, however, their stimulatory effect on gene expression of *Bace1* should be taken under consideration. In this experimental setting using AβO, the expression of *Parp1* was only slightly elevated. However, pharmacological inhibition of PARP1 activity evoked significant enhancement of gene expression of *Parp3*. Recent studies reported a particular role of PARP3 in cellular response to DNA double-strand breaks. It was described that PARP3 shares high degree of structural similarities with PARP1 and PARP2. This findings may suggest that under inhibition of PARP1, PARP3 could be responsible for the maintenance of genomic integrity, through interaction with Polycomb group proteins involved in BER/SSBR DNA repair and non-homologous endjoining (NHEJ) [69, 70].

It was previously documented that massive oxidative stress and DNA damage may lead to overactivation of DNA bound PARP(s) and to NAD^+^ reduction. Consequently, it may decrease activity of SIRT1, which is one of the positive regulators of α-secretase, a crucial enzyme in non-amyloidogenic processing of APP [71]. Concomitantly with these events, amyloidogenic pathway of APP processing may be strengthened with excessive production and accumulation of neurotoxic Aβ [72]. Inhibition of β- and γ-secretase or activation of α-secretase was suggested as promising targets in therapy of AD. Unfortunately, this type of therapeutic strategy is till now not successful [73, 74]. Some of the inhibitors and activators have entered human clinical trials, but only those targeted SIRT1 seem to be promising. Recent data about beneficial action of SIRTs focused mainly on SIRT1. Several direct and indirect activators were considered for further studies. However, the therapeutic strategy based on these compounds is till now debatable [75]. It was observed that PARP2 deficiency increased *Sirt1* transcription [76]. Also, PARP1 inhibitors were proposed to be the best activators of SIRTs. Recently, Bai et al. [77] demonstrated that PARP1 inhibition increased mitochondria metabolism through SIRT1 activation. Their data showed that PARP1 inhibition had strong effect on NAD^+^ level and other metabolic processes through modulation of SIRT1 activity which in turn enhanced mitochondria content and function. In this way, PARP1 inhibition may exert protection against metabolic, and probably also against neurodegenerative disorders. Our data indicated significant effect of PARP1 pharmacological inhibition on *Sirt1*, *Sirt6*, and *Sirt4* and *Parp3* expression levels. However, the data of Lapucci et al. [78] showed that mitochondria homeostasis is compromised in cell lines exposed to PARP1 pharmacological inhibitors or small interfering RNA. The last data emphasized the relevance of PARP1 inhibitors in therapy of mitochondrial respiratory disorders [79–82]. Molecular interaction between PARPs and SIRTs and the role of PARP1 in neurodegenerative diseases is very complex and till now not fully elucidated [31–33, 49]. Moreover, recently Szczesny et al. [83] suggested the opposing roles of mitochondrial and nuclear PARP1 in regulation of mitochondria and nuclear DNA integrity and in regulation of mitochondria function. Until now, little is known about relationship between PARPs and mitochondrial SIRTs including SIRT4. In contrast to SIRT1 and SIRT3 which possess mainly deacetylase activity, SIRT4 and SIRT5 exhibit low deacetylase activity. SIRT4 mainly performs protein mono-ADP-ribosylation and regulates ATP homeostasis and mitochondrial biogenesis [84]. SIRT5, another mitochondrial sirtuin, is capable to carry out demalonylation, desuccinylation, and glutarylation of mitochondrial proteins with almost 1000 times higher catalytic activity than deacetylation [85, 86]. SIRT5 can also modulate mitochondrial physiology and promotes respiration and ATP synthesis via desuccinylation [87]. Moreover, SIRT5 is a regulator of lysine malonylation [88]. SIRT5 also desuccinylates and activates Cu/Zn superoxide dismutase (SOD1) independent of deacetylation to eliminate reactive oxygen species (ROS) [89]. In this study, we show that short-term action of Aβ oligomers lead to activation of gene expression for *Sirt4* and *Sirt5*. However, endogenously liberated Aβ peptides in APPwt cells had no effects on mitochondrial SIRTs transcription, suggesting transient stimulatory effect of exogenous AβO. It was demonstrated previously that modulation of SIRT1 could be beneficial in AD via activation of non-amyloidogenic processing of βAPP [90]. Our results demonstrated that PARP1 inhibition activated *Sirt1* expression in the presence of AβO and upregulated gene expression of α-secretase (*Adam10*). Recently, *Sirt1* was also shown to decrease *Bace1* expression levels and β-amyloid generation in cells culture [91]. In vitro studies on human cell lines and rat primary cortical neurons have shown that γ-secretase is also regulated by SIRTs [92].

Summarizing, our results indicate that inhibition of PARP1 through the modulation of transcription of SIRTs and APP-cleaving enzymes may have significant implication on cellular processes and cell fate. In consequence, it may be important in therapy of neurodegenerative disorders.
